# Effect of sowing date and water availability on growth of plants of chia (*Salvia hispanica* L) established in Chile

**DOI:** 10.1371/journal.pone.0203116

**Published:** 2018-09-12

**Authors:** Herman Silva, Camila Arriagada, Samuel Campos-Saez, Cecilia Baginsky, Giorgio Castellaro-Galdames, Luis Morales-Salinas

**Affiliations:** 1 Laboratorio Relación Suelo Agua Planta, Departamento de Producción Agrícola, Facultad de Ciencias Agronómicas, Universidad de Chile, Santiago, Chile; 2 Laboratorio de Semillas, Departamento de Producción Agrícola, Facultad de Ciencias Agronómicas, Universidad de Chile, Santiago, Chile; 3 Laboratorio de Nutrición Animal, Departamento de Producción Animal, Facultad de Ciencias Agronómicas, Universidad de Chile, Santiago, Chile; 4 Laboratorio de Investigación en Ciencias Ambientales (LARES), Departamento de Ciencias Ambientales y Recursos Naturales Renovables, Facultad de Ciencias Agronómicas, Universidad de Chile, Santiago, Chile; College of Agricultural Sciences, UNITED STATES

## Abstract

From 2010 to 2014 two trials were performed to assess the effect of sowing date (SD1, SD2) and irrigation treatments (IT1, IT2) on the growth of chia in central Chile, measuring leaf area (LA) and dry matter (DM) as primary parameters and relative growth rate (RGR), net assimilation rate (NAR), leaf weight ratio (LWR), crop growth rate (CGR) and specific leaf weight (SLW) as secondary parameters. Both LA and DM reached maximum values between 640 and 1150 accumulated degree days (ADD). However, LA and DM were 25% greater for sowing dates than for available water. Flowering date was also not affected by sowing date or water availability; plants flowered at 1140 and 942 ADD in SD1 and SD2 respectively, and at 499 ADD in the water availability trial. Sowing date had a significant effect on RGR 0.15 g g^-1^ d^-1^ for SD1 and 0.12 g g^-1^ d^-1^ for SD2 at 410 ADD. Greater water availability increased RGR by 60% compared to stressed plants, however NAR was similar between sowing dates with a tendency to greater values in SD2 plants; maximum values were recorded at 514 ADD in IT1 and IT2, with a tendency toward higher values in IT1. Thus, the primary growth variables LA, DM and flowering are genetically determined. However, the derived growth variables RGR, LWR, NAR, CGR and SLW were affected by sowing date and water availability, with significant differences at p≤ 0.05. The results showed that the sowing date and water availability influence significantly the growth parameters. The physiological component (NAR) show a strong influence on the growth rate of the chia (RGR), above the morphological component (SLW and LAR).

## Introduction

Studies in chia have been centered on the nutritional composition of its seed, due to their high content of omega 3 (α-linolenic acid) [1], content in oil [2] and polysaccharide production in the mucilage of the seed coat [3]. Recent research has been focused on the content of natural antioxidants [4] and phenolic compounds [5] in its seeds and leaves, indicating that chia consumption would be beneficial to human health [6, 7].Chia does not tolerate frost in any development stage; the minimum and maximum growth temperatures are 11°C and 36°C respectively, with an optimal range between 16 and 26°C [8]. It has high heritability for some phenotypic characters [9] such as uniformity in its periods of flowering and maturity; [10, 8] mentioned that chia is a short-day species, flowering when nights are longer; [11] indicated a threshold between 12 and 13 hours.

In recent decades the analysis of plant growth has developed as a discipline, related to the ecophysiology and agronomy of cultivated species, with its own concepts, terms and calculation tools [11, 12]. Vegetative growth is understood as a holistic, explicative and integral explanation that permits understanding plant form and function [13, 14, 12]. Growth is determined using direct measures of primary variables (plant height, stem diameter, number of leaves, leaf area and dry weight) and indirect or secondary parameters such as the net assimilation rate (NAR) and relative growth rate (RGR), thus the growth curves of plants are a reflection of the behavior of a plant in a particular ecosystem over time. RGR can be factored into two components, the physiological and the morphological, that determine the carbon economy in the plant [15, 16]. The physiological component is the net assimilation rate (NAR) is a measure of the net carbon content [17] highly correlated with net photosynthesis [18]. The morphological component is related to the amount of leaf area (LAR) and depends on two components—the specific leaf area (SLW) and the distribution of assimilated. A combination of these growth parameters explains different yields better than any individual growth variable [19]. These determinations are indispensable for the rational application of cultivation methods at the correct moment, to guarantee optimal response of the plant according to its needs and exigencies [15]. Thus studying the ecophysiology of chia growth would contribute to the understanding of its interaction with the environment.

Agronomic research has been concentrated on the evaluation of genotypes and sowing date under different environmental conditions [8, 9], without conclusive results given the high genotype x environment interaction described in the species, which is manifested in the unpredictability of yield and grain quality due to its sensitivity to the photoperiod and low temperatures [20]. Thus [21] concluded that sowing date is relevant; it determines the length of vegetative growth, since flowering is fixed by day length. In most crops that are not sensitive to photoperiod, early sowing, associated with low temperatures and long days generates a longer duration of the growth phase, which means more radiation intercepted and thus greater RGR associated with greater IAF, as observed in wheat [22] and canola [23]. However, when chia is established under non-optimal photoperiod (long days) its development cycle is lengthened, and although it accumulates more biomass because the plants grow taller, this may generate serious stretching problems. In other situations when the photoperiod is inadequate, flowering may occur before the plant accumulates sufficient degree days (600–700°C d.), generating a low IAF and biomass accumulation [21]. There is no more information available about growth parameters in photoperiod-sensitive plants.

Water deficit is the main limiting factor for RGR, especially in semi-arid areas, limiting the growth of plants [24]. However, despite the importance of the effects of the water deficit on the RGR, the available information is limited to a few species, mostly grasses [16].Some authors have indicated that the variation in RGR is due to water deficit [12, 25] did not find significant differences in the contribution of LAR and NAR to the variation of RGR in irrigated and non-irrigated wheat plants. According to [16] there may be different effects of water deficit in the relative contribution of NAR, SLW and root development in the decrease of the RGR, In the case of annual species there may be adjustments in the SLW and root development in response to drought.

The dynamics of growth and the distribution of assimilates depend strongly on water availability and temperature as basic factors that influence life history, specifically the rate of its change of physiological time [26]. Root biomass generally increases under dry conditions, improving the chances of finding water [25,27]. Plants with high RGR may be in better condition to face stressful environmental conditions than those with low growth rate [17]; those with rapid distribution of assimilates to the leaf area may decrease water evaporation from the soil, promoting transpiration.

### Crop growth model

Many studies rely on the use of sigmoid-type mathematical relationships to characterize the evolution of crop growth over time, where the independent variable used can be days after sowing, leaf area index, luminosity or the so-called time thermal, which corresponds to the accumulation of degree-days (ADD) [28]. The concept of thermal time or degree-days is based on the hypothesis that the rate of biomass accumulation increases linearly with temperature and is directly proportional to the difference between the daily average temperature and a threshold temperature at which there would be no growth [26, 29]. Since temperature is a fundamental factor governing physiological changes in a crop, thermal time provides a quantitative estimate of those changes. This numerical approach has shown good results in the dynamic modeling of the growth and phenology of crops [30, 31, 32, 33].

### Reference evapotranspiration as a criterion for irrigation

It has been shown that the reference evapotranspiration (ETo) has a key role in irrigation scheduling. The most widely used model for estimating ETo is the Penman Monteith equation proposed by the Food and Agriculture Organization of the United Nations (FAO) [34]. This approach defines ETo as the water consumption of a reference crop that grows under optimal conditions. The Pennman Monteith model is the most used and validated method under different climatic conditions since it includes physical, aerodynamic and physiological effects. This model has been used to validate simpler models developed with the objective of obtaining ETo in areas where there are limitations of meteorological information [34, 35, 36, 37, 38, 39, 40]. Crop water requirements (ETc) are traditionally estimated using the crop coefficient (kc) according to the FAO methodological proposal [34], however, in the absence of kc data ETo may be used as an irrigation criterion [41, 42, 43]

This study is part of a project of introduction and adaptation of chia in Chile; it began with the two trials established in different sites, the first oriented to define sowing dates, including different areas between 18 and 34° S, and with this information the water availability trial was established. The mechanistic study of growth may help to understand better the acclimation of chia to diverse environments, thus promoting its expansion and commercial production. Thus, the objective of this study was to evaluate the effect of sowing date and water availability on the components that determine the growth dynamics of *Salvia hispanica* L.

## Materials and methods

The trial was performed in two growing seasons at two sites (2010–2011 and 2013–2014), the first season associated with sowing dates and the second with water availability. The 2010–2011 season was performed at the Antumapu campus of the Facultad de Ciencias Agronómicas of the Universidad de Chile, 33°40’ S, 70°38’W, at 420 m elevation. The climate of the Antumapu campus according to the Köeppen climate classification is warm temperate [43], with a prolonged dry season of 7–8 months. Mean annual precipitation is 278 mm, concentrated between May and August. The mean annual temperature is14°C; January is the warmest month and July is the coldest [44]. The soil of the Antumapu campus is of alluvial origin classified as Fluventic Haplloxerols of clay loam texture, belonging to the Santiago series [45]. The second season was performed between January and June 2014 at the Experimental Station Intihuasi (29°59’16” S, 71°17’48” W) at 135 m elevation which belongs to INIA [46], located in the Coquimbo region in northern Chile. The climate is a warm desert, with mean maximum temperature of 28°C in January and mean minimum of 5°C in July; the locality has a yearly water deficit of 800–2100 mm and 75 mm mean annual precipitation [44]. The soil, classified as Xeres Aridisols [46], is in the sandy loam texture class, with 54.8, 28.2 and 17% sand, silt and clay, respectively, an apparent density of 1.23 g cm^-3^, effective depth of 80 cm, well-drained and with low (1.2%) organic material content.

### Plant material

The chia seeds were provided by the Benexia Company, identified as lot CF3.14.9 from Santa Cruz, Bolivia, located at 17° 48’ S latitude and 63° 10’ W longitude, at 428 m elevation.

### Plant treatments

#### 2010–2011 season

The trial was performed in a surface area of 257.4 m^2^; two sowing date treatments were evaluated, sowing on 7 and December 31, 2010. The statistical design was completely randomized with 5 replicas per treatment. The experimental units were plots of 19.6 m^2^, in which we established 7 rows of 7 m length with 40 cm between rows. The observation unit was the five central rows, omitting the extremes of each plot.

The soil was initially prepared by dragging; later the seed bed was prepared with a rotovator. Soil analysis indicated nitrogen, phosphorous and potassium content of 36, 7 and 151 mg kg^-1^, respectively. Nitrogen fertilization of the crop was performed as a function of the fertility analysis and the antecedents that existed in the production of the crop [8], using a single dose of urea to provide 35 kg Nha^-1^availability in soil 15 days after emergence. Fertilizer was applied manually between rows, followed by irrigation. Seeding was performed with a manual seeder (Earthway) adapted to the small seed size, using a dose of 10 kg ha^-1^. Plants were thinned to 50 plants per m^2^ when they were approximately 10 cm tall. Weeds were controlled manually, maintaining the soil clean. Drip irrigation was used with simple tape lines, maintaining the soil in friable conditions. Irrigation frequency was determined by monitoring the soil humidity at 20 cm depth using bore holes, every two days until emergence and then every 7 days. The physical soil constants were determined using a pressure chamber; the field capacity (-33 kPa) was 19.5 g g^-1^ and the permanent wilting point (-1500 kPa) was 8.7 g g^-1^.

#### 2014 season

The sowing date was chosen based on the results obtained by Baginsky et al, [21] developed in Chile between 22° and 34° LS. Seeds were sown on 23 January 2014 in a continuous stream manually at 1 cm depth, with a seed dose of 5 kg ha^-1^. Plants were thinned between 55 to 65 plants∙m^-2^ twenty days after sowing (DAS). Soil analysis indicated nitrogen, phosphorous and potassium content of 25, 27 and 120 mg kg^-1^, respectively. Using the information obtained from the analysis of water and soil and based on the usual commercial fertilizer dosage, 30 kg ha^-1^ N and 37 kg ha^-1^ P, we used fertirrigation to apply partial doses equivalent to 20 kg monoammonium phosphate and 18 kg potassium sulfate. Weeds were controlled manually up to approximately 60 days after sowing (DAS), when the crop reached maximum cover. The physical soil constants were determined using a pressure chamber; the field capacity (-33 kPa) was 14.8 g g^-1^and the permanent wilting point (-1500 kPa) was 6.2 g g^-1^.

### Application of irrigation water

The application of irrigation water for chia plants was based on reference evapotranspiration [34], since until now the culture coefficient (kc) specific for *Salvia hispanica* is unknown. The reference evapotranspiration was calculated from the Penman-Monteith method [34] in the following manner (Eq 1):

$$ETo=\frac{0.408\cdot \Delta \cdot \left(R_{n}-G\right)+\gamma \cdot \left(\frac{900}{T+273}\right)\cdot u_{2}\cdot \left(e_{s}-e_{a}\right)}{\Delta +\gamma \cdot \left(1+0.34\cdot u_{2}\right)}$$ (1)

where ETo is the evapotranspiration of the reference crop (mm day^-1^), Rn is the net radiation on the crop surface (MJ m^-2^ day^-1^), G is the soil heat flux (MJ m^-2^ day^-1^)), T is the average temperature of the air (°C) at two meters height, μ_2_is the average daily wind speed at two meters height (ms^-1^), *e*_*s*_ is the saturation vapor pressure in kPa and *e*_*a*_ is the current vapor pressure (kPa), Δ is the slope of the vapor pressure curve *versus* temperature (kPa°C^-1^) and γ is the psychrometric constant (kPa°C^-1^). To ensure the integrity of ETo estimation by Pennman-Monteith, it is necessary to follow the recommendations proposed in FAO document 56 [3, 47, 48].

The trial consisted of two treatments and an irrigation factor with 100% (IT1), and 40% (IT2) of the reference evapotranspiration demand (ETo), calculated daily by the method of Penman Monteith [35] and summarized weekly. ETo was calculated using data from the Intihuasi monitoring and ecological modeling station located 100 m from the experimental site. To apply variable amounts of water in each treatment we used manual irrigation programming, but with automatic operation. To do this, before the trial began and with the irrigation network installed in the field we applied irrigation times of 1, 2, 3, 4 and 5 minutes using a Rain Bird ESP-LX programmer to each experimental sector, repeating the operation 6 times; all volumes were quantified using a domiciliary flow meter (precision 1 liter) in each irrigation subsector. This allowed us to associate irrigation times of the programmer with the actual number of liters discharged by the emitters in each experimental subsector (CV = 0.05 and singular losses of irrigation subsectors less than 2%). We observed the daily diurnal ETo during the experiment, using the accumulation of 5–10 mm as the irrigation criterion. To do this we transformed the accumulated ETo in mm of a period to liters per square meter and applied these liters as a function of the experimental area and the programming time, checking values with the flow meters. The irrigation network was always pressurized. We expected that the 100% ETo treatment would not generate water stress in the crop.

The original experimental design was split plots in six completely randomized blocks. In this trial the main plot was irrigation. The experimental unit was defined as a plot of 6 rows of 5 m length, with 0.6 m between rows. Irrigation treatments started at 54 DAS, at the stage of the fourth pair of leaves. Drip irrigation consisted of a tape line with a flow of 2 L h^-1^, controlled using a Rain Bird programmer (Rain Bird Corporation, Tucson USA) and maintaining independent tubing for each water treatment; each had a solenoid and flow meter to record the total water applied in m^3^. Plants were irrigated when there was a loss of between 5 to 10 mm from ETo. If the accumulated ETo in a period was 8 mm, the 100% treatment received 8 L m^-2^ (IT1) and the 40% treatment received 3.2 L m^-2^(IT2).

### Crop mathematical model

We evaluated heat accumulation in growing degree days (ADD) from sowing until the initial branching state, emission of inflorescence and flowering, summing the ADD accumulated in each time period evaluated. Growing degree days (Eq 2) were calculated with the function proposed by De Candolle [49]:

$$GDDi=\sum_{t_{i}}^{t_{f}}\left(T_{i}–T_{b}\right)\;if\;GDDi<0\;then\;GDDi=0$$ (2)

*ADDi* is the accumulated growing degree days, T_i_ is the mean air temperature on day *I* and *T*_*b*_ is the base development temperature, in this case 10°C, given the area of origin of the species [50]. Maximum and minimum daily temperatures were obtained from the records of the La Platina INIA Experimental Station, 1.5 km SE of the location of the trial in the Metropolitan Region for DS, and the records of Intihuasi INIA IV Region experimental station for IT.

The evaluations of DM and LA were performed in plants of the three central rows of each experimental unit, to avoid possible border effects. Ten plants were selected at random in each experimental unit. Every 10–15 days beginning at 24 DAS (days after sowing) in the seeding trial and at 54 DAS (once the crop was established and water treatments had begun in the water availability trial) we measured the growth of plants that were perfectly competent. Plants were cut at ground level, removing all the aerial parts. The leaves of each plant were removed individually; and their areas were determined using an electronic area integrator model LI 3000-A. The plants were dried in an oven with forced ventilation at 70°C to constant weight, then they were weighed and the dry matter (DM) of roots, leaves, stem and inflorescences were determined.

The method used to estimate the dry matter accumulation or aerial biomass as functions of time and chia phenology is based on fitting the biomass accumulation data (BIOM) using a double logistic function [51, 52, 53, 54]in the growth cycle. This method is very useful when one needs to represent the complete crop cycle quantitatively from emergence to senescence. The double logistic function has the following expression:

$$BIOM(t)=BIOM_{\min}+\left(BIOM_{\max}-BIOM_{\min}\right)\cdot (\frac{1}{1+e^{-m_{S}\cdot (t-S)}}+\frac{1}{1+e^{m_{A}\cdot (t-A)}})$$ (3)

**Fig 1:**
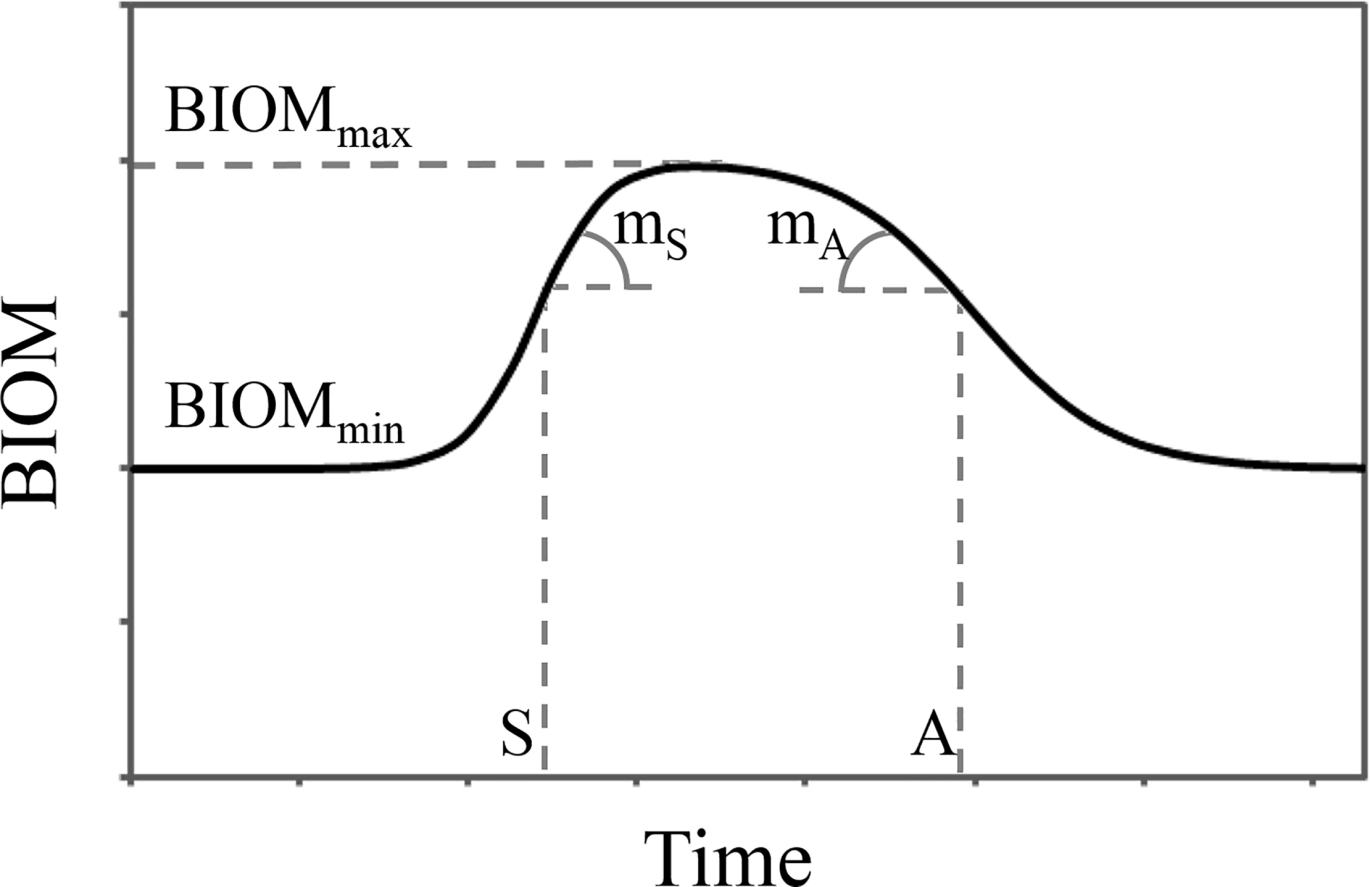
Example of the double logistic function used to model the evolution of an annual crop as a function of thermal time. This function is defined by six parameters related to emergence and the maximum potential of dry biomass accumulation (BIOM_min_ and BIOM_max_), inflection points in the growth and senescence (S and A) stage and the rates of growth and senescence S and A (m_S_ and m_A_).

BIOM (t) is the evolution of the total dry biomass from the sowing date to senescence expressed in thermal time (accumulated degree days (ADD) since crop emergence), BIOM_min_ is a value associated with the initial positive biomass of the crop, BIOM_max_ is the maximum dry biomass, S is the inflection point in the growth curve, A is the inflection point of senescence, m_S_ is related to the growth rate and m_A_ is related to the rate of senescence (Fig 1). This type of equation describes the behavior of biomass accumulation adequately for most annual crops. The fitting procedure is usually performed using the technique of Levenberg-Marquardt [55]. For leaf area (LA), the double logistic function has the following expression:

$$LA(t)=LA_{\min}+\left(LA_{\max}-LA_{\min}\right)\cdot (\frac{1}{1+e^{-m_{S}\cdot (t-S)}}+\frac{1}{1+e^{m_{A}\cdot (t-A)}})$$ (4)

FA (t) is the evolution of LA per plant from sowing to senescence expressed as ADD accumulated since crop emergence, LA_min_ is a value associated with the first positive LA value, LA_max_ is the maximum LA value, S and A are the inflection points described for Eq 1, as are m_S_ and m_A_. All the parameters of Eq 2 have an interpretation similar to that of Eq 1, according to Fig 1.

### Growth analysis

Based on the **LA** data (cm^2^) and total dry matter **DM** or BIOMASS (g)per plant, using the equations proposed by Hunt [14], we calculated the **RGR**: the rate of biomass gain per biomass(g g^-1^ d^-1^): **NAR**: rate of biomass increase per leaf area (g cm^-2^ d^-1^); **LWR**: leaf area per total dry biomass (m^2^ kg^-1^); **CGR**: increase in plant biomass per area occupied by the plant (g m^-2^ d^-1^) and **SLW:** g of leaf dry biomass per leaf area (g cm^-2^).

#### Statistical analyses

The results obtained for each locality were submitted to analysis of variance using the InfoGenprogram [56]; when significant differences were found among treatments (P < 0.05) the Duncan *post hoc* test was used to identify significantly different means.

## Results

### Thermal time and phenology

**Fig 2:**
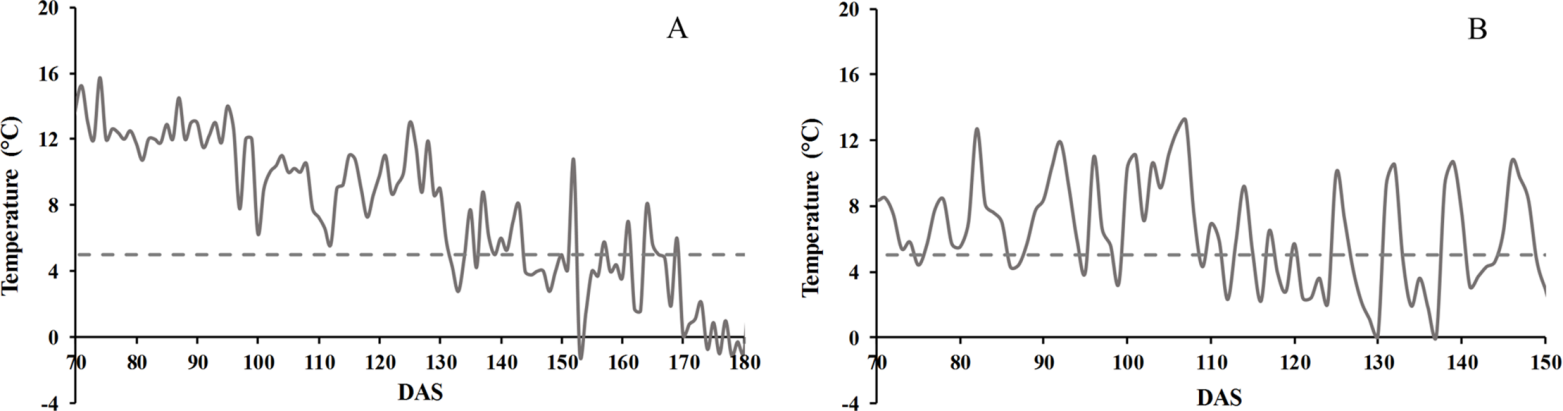
Variation of the minimum temperature from flowering to harvest in Antumapu (A, 2011) and Intihuasi (B, 2014). (DAS = days after sowing). Dashed line indicates the temperature threshold below which the plant begins to present damage.

Table 1 demonstrates the effect of sowing date and water availability associated with accumulated ADD between phenological events. Since they were sown on different dates, the 24-day displacement of SD2 resulted in less accumulated ADD from emergence to physiological maturity. However, flowering date was not related to ADD, since both flowered on the same date but at different days after sowing (DAS); 121 for SD1 and 97 for SD2. In contrast to this trial, water availability did not affect the initial days after sowing phenological stages since the treatments were begun at 54 DAS; we only found precocity in IT2 in grain formation and harvest maturity. Similar to the sowing date trial, water availability did not affect flowering date. The differences in accumulated ADD may be explained because in this study sowing date was considered to be a preliminary trial whose results allowed us to define irrigation rates, thus the ADD were lower (Table 1). Fig 2 shows the fluctuation of the minimum temperature from flowering to harvest for both trials; in Antumapu almost all days had a minimum less than 10°C with little variation, while in Intihuasi there were wide fluctuations and even days with frost.

**Table 1:** Accumulated degree days (ADD) for sowing date (SD1 and SD2) and water availability (IT1 and IT2) during the phenological development of the crop in Antumapu and Intihuasi, respectively.

Phenology Treatments	E	B	F	G	M
	ADD				
**SD1**	**43**	**301**	**1140**	**1275**	**1320**
**SD2**	**47**	**309**	**942**	**1076**	**1122**
**IT1**	**68**	**362**	**499**	**634**	**702**
**IT2**	**68**	**362**	**499**	**620**	**690**

E: Emergence, B: branches. F: flowering, G: grain filling, M: Maturity harvest

### Leaf area and dry matter

**Fig 3:**
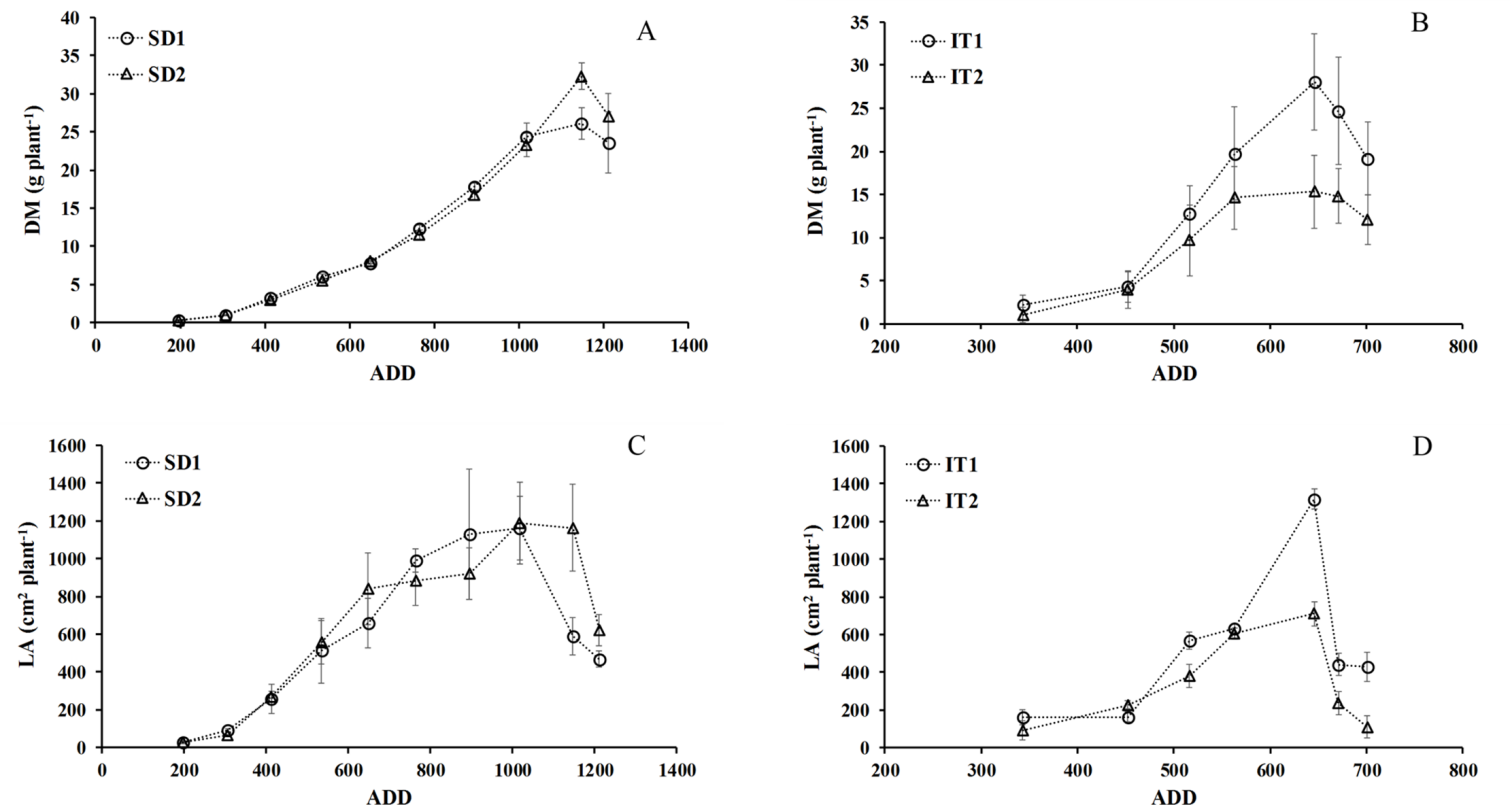
Mean dry matter production (DM) and leaf area development per plant (LA) from sowing to harvest as a function accumulated degree days (ADD). Sowing date (A, C) and water availability (B, D) in plants of chia established in Antumapu and Intihuasi, respectively.

The growth cycle of chia from sowing to harvest fluctuated from 140 to 180 days, depending upon the latitude and water availability. The maximum values of LA and DM in both trials occurred on similar dates between 117 and 122 DAS. However, there was a large difference between the trials in the ADD (Fig 3A, 3B, 3C and 3D). The lowest ADD, 600–700, occurred in the water availability trial (Fig 3B and 3D), while the sowing date trials had values from 1000–1200 ADD, since we knew the best date for sowing and establishment of the crop at this latitude. The greater accumulation of dry matter in the sowing date trial is explained by the longer growth cycle, reaching 181 days. This was excessive growth, especially for stems, which reached 180 cm at the end of the crop cycle, compared to the maximum of 150 cm in the water availability trial. This lower height was compensated by greater development of LA (Fig 3D).

During vegetative growth the sowing date did not modify the development of LA substantially (Fig 3A); it had a mean rate of 14.8 cm^2^ d^-1^ from 35 to 105 DAS. The maximum development of LA was 1187 cm^2^ and 1160 cm^2^ per plant for SD2 and SD1, respectively, at 105 DAS. SD2 plants maintained the maximum leaf area, while in SD1 there was increasing defoliation that produced a significant (p≤0.05) difference (Fig 3A), with a mean rate of 50 cm^2^ d^-1^ for SD2 and a rate of decrease of LA for SD1 plants equivalent to 25 cm^2^ d^-1^. Greater water availability (IT1) produced an LA up to 50% greater than in IT2 (Fig 3B). Even though the trials were different at sowing times, localities and soil-climate conditions, there was no significant difference in total DM between SD1 and IT1 (Fig 3A and 3B); the non-significant difference was produced by water availability, with a significant difference from the beginning of water treatments to harvest (Fig 3B). Total MS was similar between IT1 and the two sowing dates, with yield SD of about 30 g per plant (Fig 3A and 3B). However, there was a significant effect in the IT2 plants, which were significantly different beginning 21 days after the beginning of treatments (75 DAS) which was maintained until harvest (Fig 3B).

**Fig 4:**
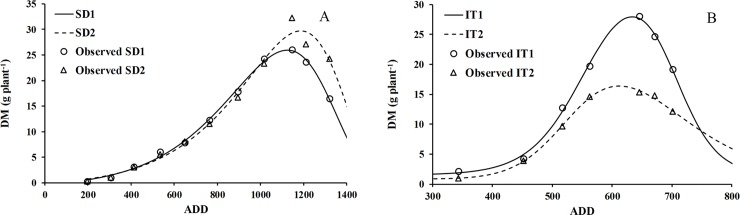
Evolution of the dry biomass (g plant^-1^) during the chia crop cycle (points). Curves were adjusted using a double logistic function (lines), as a function of accumulated degree days (ADD) for sowing date (A) and water availability (B) in chia plants established in Antumapu and Intihuasi, respectively.

Table 2 shows the estimated values of the coefficients of Eq 1, adjusted for the two trials oriented to biomass accumulation, modifying sowing date and irrigation treatment. The table shows that the most contrasting coefficients were S and A, which indicate two ADD whose rates reflect contrasting physiological phenomena—growth and senescence. The fits for both situations were significant (P≤ 0.001), with high coefficients of determination (> 0.98) and small mean square errors. Fig 4 shows the fit of the double-logistic function, which described the dry mass accumulation in the cropadequately for the sowing date (Fig 4A) and irrigation (Fig 4B) trials. These Fig 4A and 4B also reproduce the effect of crop senescence up to grain harvest, when the trials finished.

**Table 2:** Estimated values of the coefficients of Eq 3 for the sowing date (SD) and irrigation (IT) treatments oriented to biomass accumulation.

Treatments	BIOM_mix_	BIOM_man_	m_S_	S	m_A_	A	RMSE	Error	R^2^	P
SD1	-67.742	66.620	-0.0041	1086.756	0.0088	1338.448	0.4906	0.542	0.997	0.000
SD2	-103.846	102.889	-0.0039	1267.273	0.0085	1414.806	1.2865	1.412	0.986	0.000
IT1	-35.275	36.823	-0.0213	558.557	0.0289	704.103	0.485	0.573	0.997	0.000
IT2	-29.563	30.584	-0.0209	530.571	0.0121	665.970	0.243	0.287	0.998	0.000
Average SD	-85.794	84.754	-0.004	1177.015	0.009	1376.627	0.889	0.977	0.992	0.000
Average IT	-32.419	33.703	-0.021	544.564	0.020	685.036	0.364	0.430	0.998	0.000

**Fig 5:**
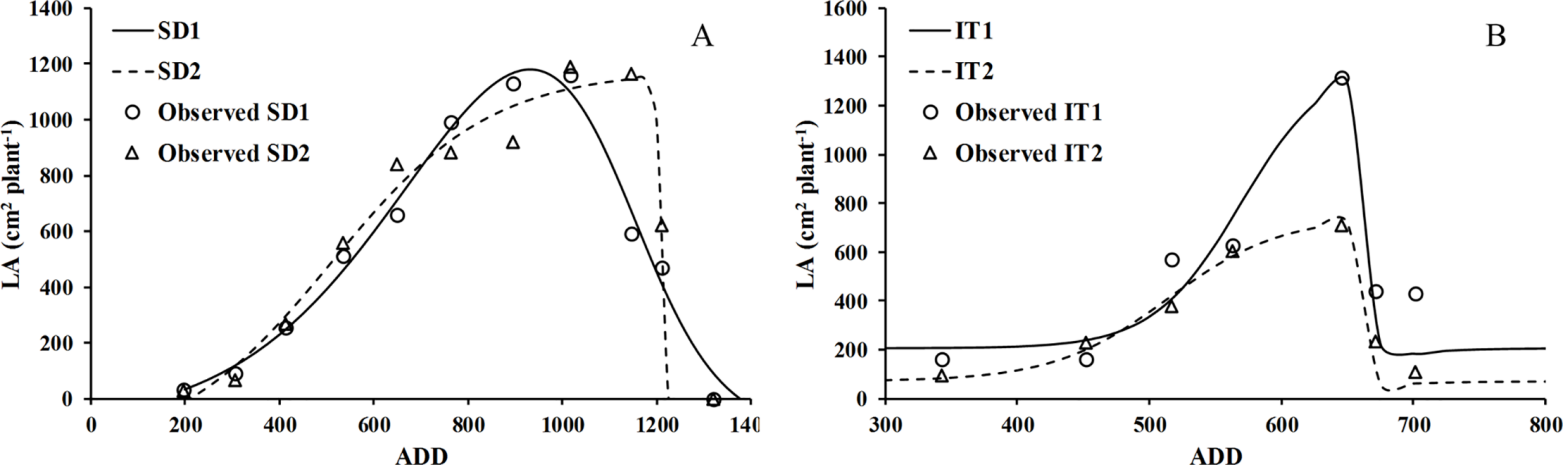
Evolution of leaf area (cm^2^ plant^-1^) throughout the chia crop cycle (points). Curves were adjusted using a double logistic function (lines) as a function of accumulated degree days (ADD) for sowing date (A) and water availability (B) in chia plants established in Antumapu and Intihuasi, respectively.

Table 3 gives the estimated values of the coefficients of Eq 2, adjusted for two trials oriented to leaf area, modifying sowing date and irrigation treatment. It shows that the most contrasting coefficients were the minimum and maximum leaf area, LA_min_ and LA_max_. The fits for both situations were significant (p < 0.001), with high coefficients of determination (> 0.95) and small mean square errors. Fig 5 shows the fit of the doublelogistic function, which describes the evolution of leaf area in the cropadequately for sowing dates (Fig 5A) and the irrigation treatments (Fig 5B).

**Table 3:** Estimated values of the coefficients of Eq 4 for sowing date (SD) and water availability (IT) treatments for leaf area.

Treatments	LA_mix_	LA_man_	m_S_	S	m_A_	A	RMSE	Error	R^2^	P
SD1	-2109.352	2009.767	-0.0051	721.877	0.0101	1142.224	45.783	50.774	0.986	0.000
SD2	-1573.041	1373.831	-0.0059	511.217	0.1472	1214.244	60.966	67.375	0.977	0.000
IT1	-979.047	1185.154	-0.0303	569.297	0.4217	668.279	136.955	147.663	0.871	0.000
IT2	-604.942	674.837	-0.0232	513.865	0.9289	669.912	35.809	40.872	0.977	0.000
Average SD	-1841.197	1691.799	-0.005	616.547	0.079	1178.234	53.375	59.074	0.981	0.000
Average IT	-791.995	929.996	-0.027	541.581	0.675	669.096	86.382	94.267	0.924	0.000

We perfomed t tests to compare the efficiency of Eqs 2 and 3 using the coefficients shown in Tables 2 and 3, which are the coefficients estimated by minimum squares for the equations with different sowing dates (SD1 and SD2) and irrigation treatments (IT1 and IT2) (Figs 4 and 5). No significant difference was found for the coefficients used for total dry weight (SD1, SD2, IT1, IT2) in the equations of Table 2 (P = 0.586), with 95% confidence. The mean of the coefficients adjusted for sowing date (SD1 and SD2) and irrigation treatment IT1 was not significant (P = 0.6). However, there was a significant difference (P ≤ 0.05) for IT2. This suggests that the coefficients found for Eq 3 are only applicable for an appropriate sowing date.

### Growth functional analysis

#### Relative growth rate (RGR)

**Fig 6:**
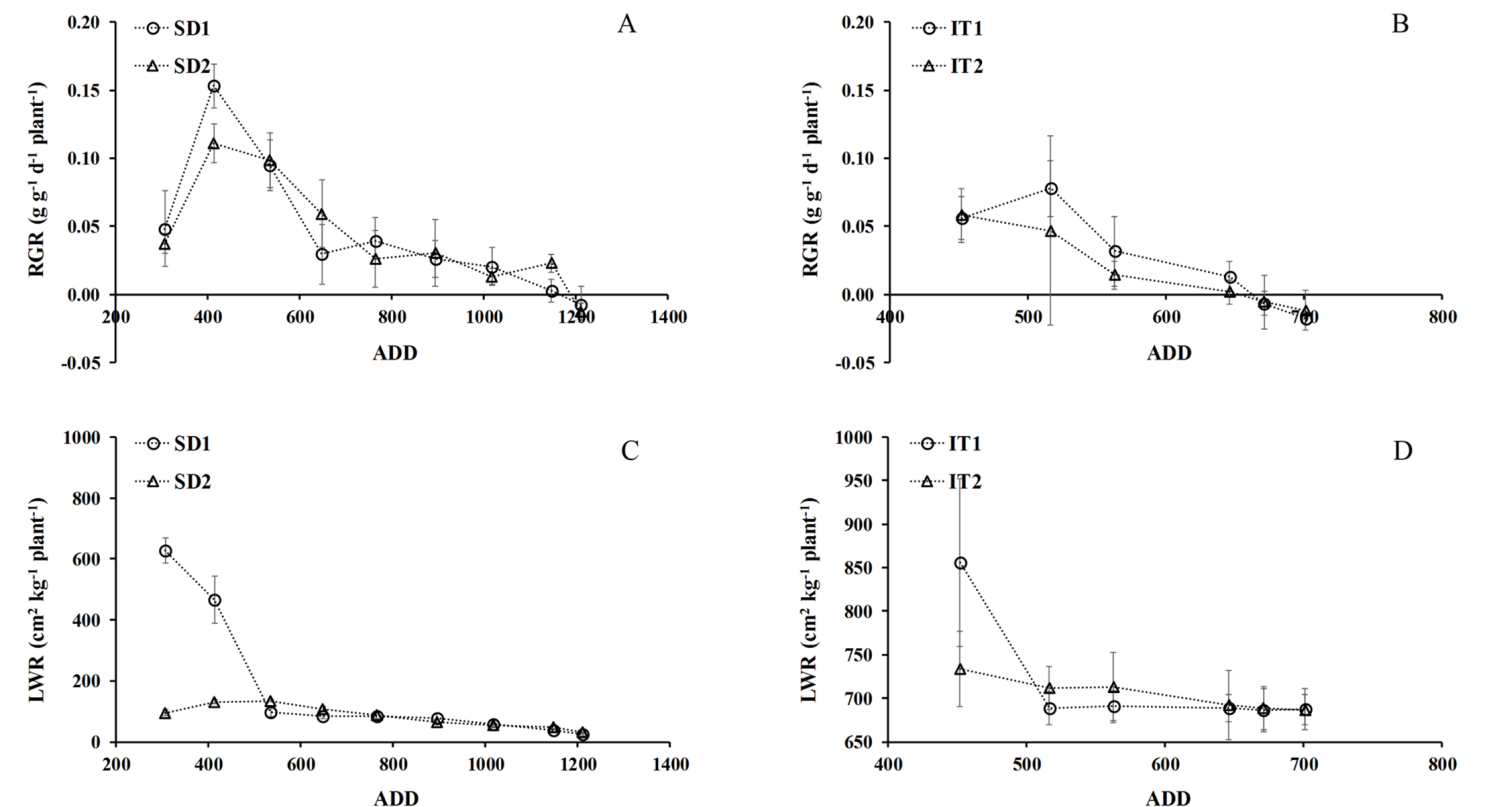
Relative growth rate (RGR) and leaf weight ratio (LWR) per plant as a function of accumulated day degrees (ADD). It shows both treatments from sowing to harvest for date of sowing (A, C) and water availability (B, D) in chia plants established in Antumapu and Intihuasi (x ± SD, n = 12).

Both sowing dates had highest RGR values (Fig 6A) at 410 ADD at aproximately 35–45 days after sowing; these were significantly different (p≤0.05); 0.15 gg^-1^d^-1^ in SD1 and 0.11 gg^-1^d^-1^ in SD2. Both decreased gradually until harvest. The maximum RGR in the water availability trial only reached 50% of those in the sowing date trial, at 514 ADD (Fig 6B) (treatments began at 54 DAS). In the water availability trial there was rapid depletion of the assimilates of the total cycle from sowing to harvest (151 days), while in the sowing dates trial the assimilates were used gradually, with a total cycle of 181 days.

#### Leaf weight ratio (LWR)

As expected, the maximum rates of leaf area development were found at the beginning of the vegetative cycle, which is asociated with the maximum RGR (r^2^: 0.6 P≤ 0.05) in the exponential growth phase of chia. In the sowing date trial the LWR in SD2 reached the highest value, 650 cm^2^ kg^-1^ plant ^-1^,probably associated with the better sowing date for this locality up to 55 DAS, while SD1 was much lower, only reaching 17% of the LWR of SD2 (Fig 6C). These values were different than those found in the water availability trial, since LWR reached 856 cm^2^ kg^-1^ p^-1^ between the beginning of treatments at 450 to 520 ADD, decreasing only to 686 cm^2^ kg^-1^ plant^-1^up to harvest (Fig 6D). In the irrigation treatments (IT) the LWR values were significant and positively related to RGR (r^2^ = 0.92), as opposed to the sowing date (SD).

The maximum value of LAI (values not shown) was 8.5 for SD1 and SD2 (difference not significant) was recorded at 122 days, coinciding with the maximum leaf area and with an ADD of 1000. In the trial of water availability the maximum values were 4.3 and 4 for IT1 and IT2 respectively, at 650 degree days, which were significantly different (P≤ 0.05).

#### Net assimilation rate (NAR)

**Fig 7:**
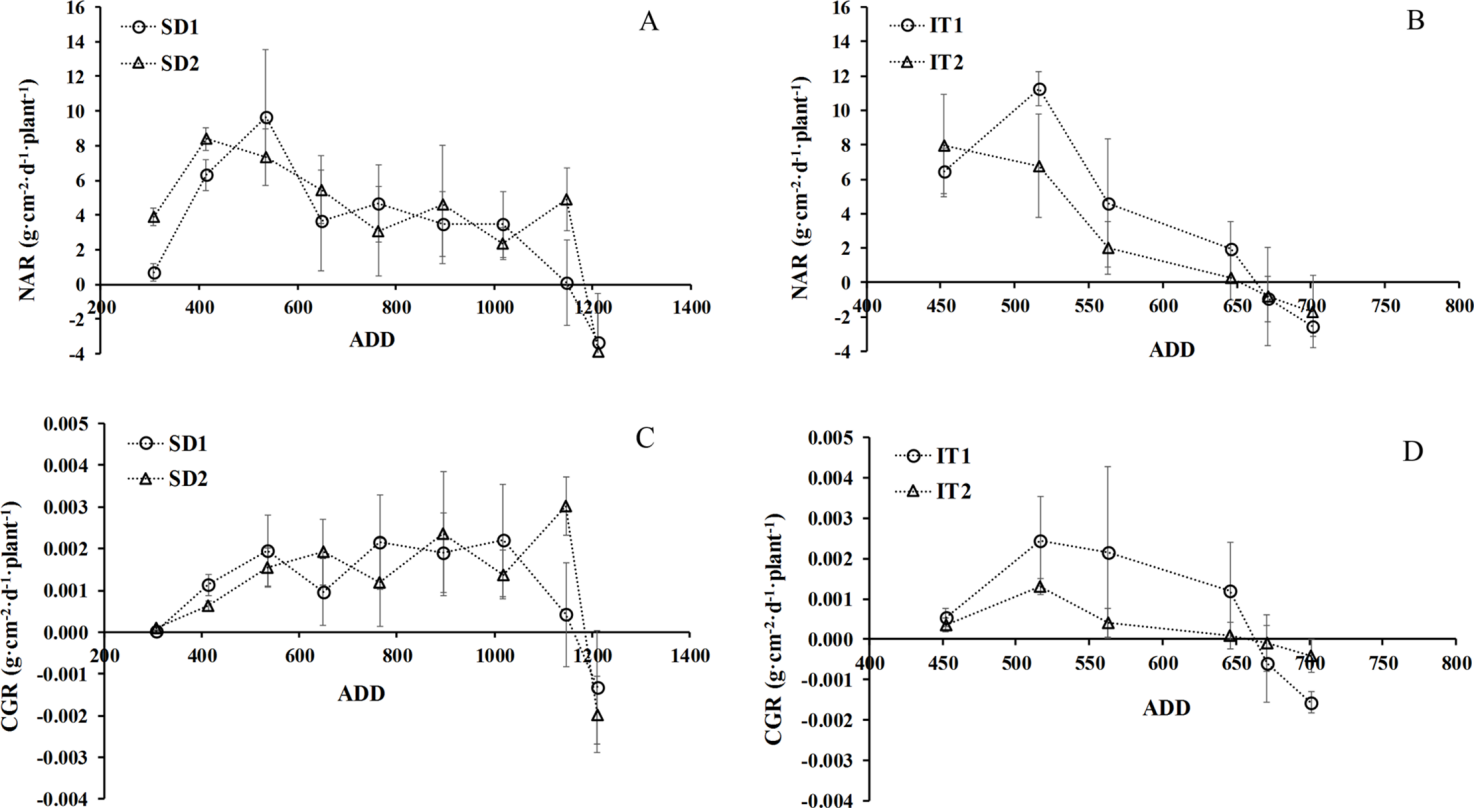
Net assimilation rate (NAR) and crop growth rate (CGR) per plant as a function of accumulated thermal time (ADD), showing both treatments from sowing to harvest for date of sowing (A, C) and water availability (B, D) in chia plants established in Antumapu and Intihuasi (x± SD n = 12).

In the sowing date trial the NAR showed similar behavior between trials from sowing to harvest, with maximum values between 8 and 10 g m^-2^ d^-1^ for SD1 and SD2, respectively, during the initial growth phase when plants were small and all leaves were exposed to radiation. Thus there were no significant differences due to sowing date or water treatments (Fig 7A and 7B); the maximum value was found at about 550 DDG in both trials, although there were significant differences in the treatments. There was a significant difference between sowing dates at 45 DAS and a tendency to greater NAR in the IT1 plants (Fig 7B). The net assimilation rate decreased throughout the cycle until the senescence.

#### Crop growth rate (CGR)

Fig 7C and 7D show the crop growth during the growing cycle for trials and treatments. Crop growth rate values fluctuated between 0 and 0.0025 and 0.0030 g DM cm^-2^d^-1^ (equivalent to 25 and 30 g m^-2^ d^-1^ in both trials, with asignificant effect of sowing date and water availability during most of the chia growth cycle but a tendency to higher values in the IT1 plants (P ≤ 0.05) (Fig 7C and 7D). Given the lengthening of the growth phase, the maximum value at sowing date coincides with the maximum values of leaf area and dry matter at 1100 ADD, unlike the water availability trial in which the maximum values were recorded in the initial phase of growth with ADD of 520.

#### Specific leaf weight (SLW)

**Fig 8:**
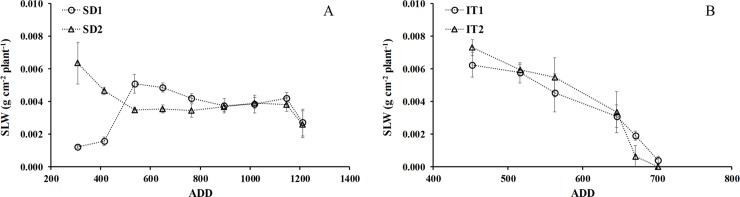
Mean specific leaf area (SLW) per plant as a function of accumulated thermal time (ADD), showing both treatments from sowing to harvest for sowing date (A) and water availability (B) in chia plants established in Antumapu and Intihuasi (x± SD n = 12).

The gap in sowing date was determinant in the exponential growth phase in SD2, associated with more favorable climatic conditions, especially temperature, and probably closer to the optimal photoperiod (Fig 8A). The growth rate was initially slow in SD1; the exponential growth phase was reached after 410 ADD (Fig 8A). From the beginning of treatments (Fig 8B) at 400 ADD (54 DAS) the value of SLW was similar after 450 ADD (60 DAS) with a significant difference (p≤0.05) only at the end of the reproductive cycle when the rapid leaf fall affected the SLW. The SLW values in SD2 were very similar to the irrigation treatments (IT).

#### Comparisons intraspecific

All the functional growth parameters used (LA, DM, RGR, LWR, NAR, CGR and SLW) were compared using the simple correlation matrix. From the functional variables analyzed, high and positive correlations with RGR were found for NAR and SLW for the irrigation treatments (IT1 and IT2), with values between 0.923 and 0.910. For the case of IT1, the correlations of RGR with NAR and SLW were 0.993 and 0.953 respectively, and 0.923 between NAR and SLW; in all cases the P value (< 0.01) was significant. This trend was also observed in the case of IT2, finding also high and significant correlations of RGR with NAR and SLW of 0.999 and 0.908 respectively, and 0.910 between NAR and SLW. In the case of the sowing dates (SD1 and SD2) a somewhat different behavior was observed between the functional parameters. For SD1, the correlation of RGR with NAR and SLW was 0.557 and -0.431, respectively, and 0.358 between NAR and SLW; in all cases the p values were > 0.1, considered non-significant. The above situation is interpreted as an inappropriate date for the cultivation of chia. For the case of SD2, a trend similar to IT1 and IT2 was observed, however, the correlation coefficients were lower. Specifically, the correlation of RGR with NAR and SLW was 0.869 and 0.276 respectively, and 0.393 between NAR and SLW; the relationship was only significant between RGR and NAR. These values, higher than those obtained in SD1, indicate that we are close to the optimum sowing date. For the case of IT2, finding also high and significant correlations of LAR with NAR and RGR of 0.8 and 0.942 respectively. These results show an influence of the physiological component (NAR) on the relative growth rate (RGR), above the morphological component (SWR and LAR).

## Discussion

We selected the double logistic curve because it shows good agreement with the data measured in the field, better than other functions such as the Fourier series, the asymmetric Gaussian functions and double Gompertz [51]. The adjustment made for the dry matter (DM) and the foliar area (LA) by means of the double logistics equation proved to be an adequate function to describe these growth parameters over time. The emergence, physiological maturity and senescence phases are clearly observed in Fig 4 for irrigation treatments and planting date. These results are similar to those obtained in other studies, which shows that this type of adjustment is a good descriptor of the growth evolution of a plant [52, 53, 54, 55].

### Sowing date

The effect of sowing date on growth has been widely documented in many species, however it has only been documented in chia in a few studies especially oriented to yield [23, 9]. Sowing date is determinant in the growth, development and yield of cultivated species, especially when dealing with the introduction and/or adaptation of a species to habitat different from its native environment. In these conditions there may be significant changes in growth parameters and those defined as primary (LA and DM) and those associated with the functional growth of the species as secondary parameters (RGR, NAR, CGR, LWR, SLW). In the trial established at 34° south latitude there were no significant differences in LA or DM from sowing until flower initiation, but differences were produced from flowering to harvest, where SD1 achieved lower values associated with greater defoliation as a result of the lower interception of light that caused the greater growth of stems and branches at this planting date (data not shown). This is a characteristic behavior of plants sensitive to short-day photoperiod such as chia, which if set in long day conditions will generate greater vegetative growth associated with a larger number of nodes and leaves and greater height until the conditions of light are those required to flower [57, 21]. In the sowing date trial, the functional growth parameters showed maximal expression from 45–55 DAS associated with photosynthetic activity; photosynthesis rates reached 25 μmol CO_2_ m^-2^ s^-1^, with maximum values of instantaneous water use efficiency in terms of intrinsic efficiency [43]. The advantage of achieving a high RGR in the early stages of crop growth (≈ 400 ADD, ≈ 45 days from sowing) is associated with a greater increase in the size of the plants that allows them to cover the soil quickly (above and below the surface)) and thereby take better advantage of water resources, nutrients and solar energy collection [58, 59, 60]. A similar trend was recorded by Van Iersel [61] in a species of the same genus *Salvia* (*S*. *splendens* F. Sellow ex Roem and Schult.), where the plants showed a maximum RGR of 0.24 g ∙ g^-1^ ∙ d ^-1^ at 15 days after sowing, reducing to 0.06 g ∙ g^-1^ ∙ d^-1^ at 70 DAS. Over time and to the extent that the plant was accumulating day degrees the RGR was decreasing, which is due to the fact that at the beginning of the crop growth cycle all the cells are involved in the photosynthetic function and production of assimilates. But as time passes, the basal leaves, being older, are not able to photosynthesize appropriately and therefore the proportion of assimilates that go to the total dry weight could decrease (therefore the proportion of assimilates to total dry weight would decrease). This process occurs because the oldest leaves are involved in the measurement of total dry weight, but they do not have a function in assimilated production [62]. The maximum biomass production efficiency in the crop cycle was at 1150 ADD in SD2, when the maximum LA and total DM values were recorded. Crop growth rate (CGR) is low at early growth stages because the plant cover is incomplete and the plants intercept and absorb only part of the solar radiation. Growth rate is quickly increased during development because of the expansion of leaf area and less radiation penetrating through plant cover to the soil surface [23]. Maximum CGR occurred when plants were sufficiently high or dense to probe all the environmental factors. Maximum CGR occurs when the leaf cover is complete in favorable environments, and may represent the maximum production potential of dry mass and conversion rates at a given moment. This is valid for SD at 122 DAS, where the maximum LAI and CGR rates were found; this coincides with the report of Lee and Heuvelink [34], where LAI increased with crop growth, reaching a maximum value in which the maximal capability of intercepting solar energy was reached, when CGR is also maximum. On the other hand, SLW, which is a way of expressing leaf area development rates [63], showed that DS2 would be more favorable for the vegetative growth of the species, which would allow a greater accumulation of leaf assimilates; this is the most important sink, corroborated by the maximum value of LAI (value not shown). The high values of LWR at the beginning of crop development en SD1 (Fig 6C) are due to the plants using more of their photoassimilates for development and growth of the photosynthetically active areas, generating energetic expenditures that result in lower weight [43]. The decrease of LAR in both trials is due to the effect of a decrease in the thickness of the leaves as the plants advanced in their development until reaching senescence, as has been observed in amaranth subjected to different planting densities and humidity levels [64]. Similar results to SD2 were obtained in the trials of [65] in four varieties of potato (*Solanum tuberosum* L.), where maximum values of about 130 cm^2^ g^-1^ were recorded at 4 weeks, which then decreased; the minimum value of approximately 20 cm^2^ g^-1^ was reached in the last evaluation at 18 weeks.

The second planting date was superior in physiological index compared to the first, achieving maximum dry matter (≈ 30 g plant^-1^), crop growth rate (30 g m^-2^ d^-1^), net assimilation rate (10 g m^-2^ d^-1^), relative growth rate (0.064 gg^-1^ day^-1^) and specific leaf area (≈ 0.0064 g cm^-2^ plant^-1^), this planting date generated higher grain yields (data not shown) due to an anticipated frost during the harvest phase that did not allow obtaining yield.

### Water availability

The water deficit decreased growth, producing a 54% decrease in the total dry matter as well as a 43% decrease in the development of the foliar area in comparison to irrigated plants (Figs 4 and 5) in the period between 300 to 700 ADD. According to Kozlowsky et al. [66] this decrease may be related to the ability of plants to tolerate water deficit through plastic morphogenetic responses. In addition to physiological responses, Silva et al. [67] showed in chia that plants subjected to water deficit have the capacity for osmotic adjustment without significant changes in the modulus of elasticity, responses associated with the maintenance of cellular turgor, on which all the processes associated with growth depend. The same author pointed out the reduction of gaseous exchange in terms of photosynthesis, transpiration and stomatal conductance, responses to the water deficit that contribute to increase the efficiency in the use of water and at the same time maintain its yield in seed [42].

The strong positive relationship found between RGR and SLW (P≤0.001), as in most of the available studies [68], is based on the fact that SLW is the main morpho-anatomical factor associated with the intraspecific variation of RGR. According to [69] low SLW is associated with a greater foliar mass, however the anatomical studies carried out in chia (data not shown) show the inverse relationship high values of SLW that are associated with greater leaf mass, which compensates for the smaller leaf area in plants subjected to water deficit. Low SLW would be associated with thinner leaves and greater availability of water; it would be inversely proportional to the internal conductance of CO_2_ which would increase the carboxylation and therefore produce greater photosynthetic activity. SLW is one way of estimating photosynthetic efficiency, as the production of dry matter per unit of leaf surface is related to greater thickness of the leaves. The greater thickness is also due to an increase in cell layers, which implies that vegetative growth is associated with greater leaf thickness per unit of leaf area, thus tending to greater photosynthetic efficiency in the rest of the crop cycle.

The relationship of RGR with LWR was not significantly positive in plants of IT1, while in stressed plants it was highly significant (P≤0.001), probably explained by the lower development of DM in relation to leaf area. It is known that the optimum LAI is that which produces the maximum rate of dry matter; this is achieved when the crop intercepts virtually all the available radiation [36], and as a result the lowest leaves are still capable of maintaining a positive carbon balance [33]. NAR was determinant in the growth of plants in the early phenological stages, when the effect of self-shading did not influence photosynthesis. This high proportion of leaf biomass at the beginning of flowering is associated with a high respiratory rate [16], which suggests that NAR is the main physiological component in the determination of RGR.

Neither sowing date nor water availability modified the date of flowering; the light requirement was 13.5 light hours [9]. The thermal time for this phenological phase in sowing date trial was 1140 and 942 ADD for SDI and SD2, respectively, and 499 ADD in the water availability test. These differences between trials and treatments could be explained by the high temperatures (day and night) which prolonged the crop cycle. According to [65], high temperatures extend the period of leaf formation leading to high vegetative growth at the expense of reproductive growth. A decrease of 2–3°C produces significant changes in the growth rate. Salisbury and Ross [70] suggested that a temperature change of 0.2 to 1°C often produces rapid growth, since this increase multiplies the speed of many biochemical reactions several-fold.

## Conclusions

The growth pattern shown by *Salvia hispanica* for dry matter (DM) and leaf area (LA) fit a double logistic curve appropriately appropriately. The results of this study allowed us to estimate a number of functional parameters in the growth of chia as a function of different trials performed in different localities. Sowing date did not affect the parameters of vegetative growth especially up to flowering. In contrast, lower water availability significantly affected the growth parameters. However, many of the responses were similar in terms of development associated with phenological changes. The duration of the vegetative cycle was dependent on water in both trials, sowing date and water availability.

## Supporting information

S1 File A compressed file that contains supplementary material about information on meteorological data, dry matter and leaf area, used in the article. (RAR)
